# Practical approach for pretreatment verification of IMRT with flattening filter‐free (FFF) beams using Varian portal dosimetry

**DOI:** 10.1120/jacmp.v16i1.4934

**Published:** 2014-01-08

**Authors:** Soonki Min, Young Eun Choi, Jungwon Kwak, Byungchul Cho

**Affiliations:** ^1^ Department of Radiation Oncology Asan Medical Center, University of Ulsan College of Medicine Seoul Korea

**Keywords:** IMRT, VMAT, EPID, flattening filter‐free beam, portal dosimetry

## Abstract

Patient‐specific pretreatment verification of intensity‐modulated radiation therapy (IMRT) or volumetric‐modulated arc therapy (VMAT) is strongly recommended for all patients in order to detect any potential errors in treatment planning process and machine deliverability, and is thus performed routinely in many clinics. Portal dosimetry is an effective method for this purpose because of its prompt setup, easy data acquisition, and high spatial resolution. However, portal dosimetry cannot be applied to IMRT or VMAT with flattening filter‐free (FFF) beams because of the high dose‐rate saturation effect of the electronic portal imaging device (EPID). In our current report, we suggest a practical QA method of expanding the conventional portal dosimetry to FFF beams with a QA plan generated by the following three steps: 1) replace the FFF beams with flattening filtered (FF) beams of the same nominal energy; 2) reduce the dose rate to avoid the saturation effect of the EPID detector; and 3) adjust the total MU to match the gantry and MLC leaf motions. Two RapidArc plans with 6 and 10 MV FFF beams were selected, and QA plans were created by the aforementioned steps and delivered. The trajectory log files of TrueBeam obtained during the treatment and during the delivery of QA plan were analyzed and compared. The maximum discrepancies in the expected trajectories between the treatment and QA plans were within 0.002 MU for the MU, 0.06° for the motion of gantry rotation, and 0.006 mm for the positions of the MLC leaves, indicating much higher levels of accuracy compared to the mechanical specifications of the machine. For further validation of the method, direct comparisons of the delivered QA FF beam to the treatment FFF beam were performed using film dosimetry and show that gamma passing rates under 2%/2 mm criteria are 99.0%–100% for the all four arc beams. This method can be used on RapidArc plans with FFF beams without any additional procedure or modifications on the conventional portal dosimetry of IMRT and is, therefore, a practical option for routine clinical use.

PACS numbers: 87.53.Kn, 87.55.T‐, 87.56.bd, 87.59.‐e

## I. INTRODUCTION

Patient‐specific pretreatment quality assurance (QA) for intensity‐modulated radiation therapy (IMRT)^(1)^ is strongly recommended for all patients to identify any potential errors in the treatment planning process and in machine deliverability, and thus is performed in many clinics to ensure accurate delivery of IMRT, including volumetric‐modulated arc therapy (VMAT).^1^, ^2^

Two‐dimensional array detectors are widely used for IMRT QA, but may be inappropriate for pretreatment verification of SBRT, which usually involves field sizes smaller than $5\times 5\;{\text{cm}}^{2}$ due to its lower spatial resolution that results in coarse sample size insufficient for adequate gamma analysis.

As an alternative, portal dosimetry utilizing an electronic portal imaging device (EPID) is an attractive method for routine use because of its prompt setup, easy data acquisition, and high spatial resolution.^(3)^ Varian Portal Dosimetry (Varian Medical System, Palo Alto, CA) is efficient for routine clinical use for IMRT QA due to its incorporation of an Eclipse treatment planning system and amorphous silicon (aSi) EPID. Furthermore, there have been recent advances in Varian Portal Dosimetry, including optimization of the dosimetric response of the aSi imager with incorporation of 2D profile and backscatter corrections.^4^, ^5^

However, portal dosimetry has a maximal dose rate limit of 600 monitor units per minute (MU/min) due to the saturation effect of the aSi imager.^(6)^ Therefore, this technique is not applicable to flattening filter‐free (FFF) beams, which have a dose rate up to 1400 MU/min for 6 MV FFF (6XFFF) beams and 2400 MU/min for 10 MV FFF (10XFFF) beams. A recent study^(7)^ showed that this limitation for pretreatment QA of VMAT with FFF beams could be resolved by increasing the source‐to‐imager distance (SID) to 150 cm. However, the official release of the TrueBeam system prohibits the use of portal dosimetry for FFF beams in order to prevent misuse with high dose rates of the FFF beams. Therefore, cumbersome procedures, such as transferring and processing of the measured image data, are mandatory if third party applications are used.

In our current report, we suggest a practical QA method of expanding the conventional portal dosimetry to FFF beams with a QA plan generated by the following three steps: 1) replace the FFF beams with flattening filtered (FF) beams of the same nominal energy; 2) reduce the dose rate to avoid the saturation effect of the EPID detector; and 3) adjust the total MU to match the gantry and MLC leaf motions. Our approach can be used without any additional procedure or modifications to the conventional portal dosimetry of IMRT, and thus may be more practical in routine clinical use.

## II. MATERIALS AND METHODS

Two stereotactic body radiation therapy (SBRT) cases treated by a TrueBeam machine were selected for our present analysis. RapidArc plans composed of dual‐arc 6XFFF beams and 10XFFF beams were generated using the Eclipse treatment planning system (Ver. 10). The maximum allowed dose rate of the beams was set at 1200 MU/min due to the unclear *in vivo* biological effect associated with high‐dose‐rate photon beams.^(8)^

### A. Portal dosimetry

IMRT QA was performed using the Varian Portal Dosimetry application (Ver. 10) by comparing the predicted dose images calculated from the Eclipse treatment planning system (TPS) with the measured dose images acquired from the aSi imager. A Portal Vision aS1000 imager panel of TrueBeam linac (Ver. 1.5) was used, with a pixel dimension and spatial resolution of 1024×768 and 0.392 mm per pixel, respectively.

Dosimetry calibration of the EPID imager is required for the acquisition of portal dose images. The first step of dose calibration consists of beam profile correction for nonuniform X‐ray beam intensity that is assumed during dark field and food field imager calibration. The second step is dose normalization, which relates the measured signals to the radiation dose using a reference condition, normally defined as the calibration unit (CU) by the digital signals per MU. After dose normalization, the measured dose images are generated by converting the EPID signals into the calibrated units (CU), which can then be compared to the predicted dose images. All calibrations and measurements were performed at a source‐to‐imager distance of 100 cm.

### B. QA plan

**Figure 1:**
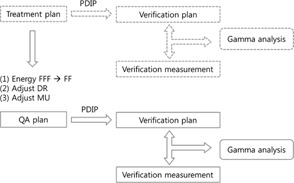
Flowchart comparing the procedures of the normal portal dosimetry for IMRT QA (dashed line) to those of the method proposed in this study (solid line). The portal dosimetry procedure of flattening filtered (FF) beam involves a creation of verification plan with a portal dose image prediction (PDIP) to compare with verification measurement of aSi imager via gamma analysis. Since direct use of portal dosimetry is not available for flattening filter‐free (FFF) beam, a QA plan is generated from the original treatment plan by replacing FFF beam with FF beam, and adjusting dose rate (DR) and total monitor unit (MU) while ensuring the same beam delivery machine parameters including gantry speed and leaf motions.

The original treatment plan was a dual RapidArc fields with 6XFFF beams or 10XFFF beams. As shown in Fig. 1, since the original treatment plan with FFF beams cannot be directly used for portal dosimetry due to the saturation of the EPID panel at high dose rates, a QA plan was generated from the original treatment plan which can be used for portal dosimetry, while ensuring the machine delivery parameters are unaffected. A QA plan was created by first copying each 6XFFF (or 10XFFF) treatment plan, then replacing the energy of the FFF beam with the same nominal energy of the flattening filtered (FF) beam (e.g., 6XFF (or 10XFF)). Finally, to match the speeds of gantry rotation and MLC motion of the QA plan to those of the actual treatment plan, the dose rate of the QA plan was set at the maximum dose rate of the FF beam, 600 MU/min, and the total MU of the QA plan was subsequently reduced in the same proportion as the dose rate reduction.

### C. Gamma analysis

After the QA plan was prepared, a verification plan for portal dosimetry was made from the QA plan using the Eclipse treatment planning system, which computes the predicted dose image for comparison to the measured portal dose image (Fig. 1).

Gamma analysis is well established as a method of quantitatively comparing dose distributions, either measured or calculated.^(9)^ In our present analysis, dose distributions were compared in an absolute mode, where the predicted and measured CU values are directly compared after normalizing 100% to the maximal predicted CU. Only those pixels above 10% of the maximal predicted CU were included in the analysis. The gamma index was computed using acceptance criteria of 3%/3 mm. Furthermore, gamma passing rates, which are the percentage of pixels whose gamma index is less than 1, were also computed.

In addition, the mean absolute dose difference between the predicted and measured doses for the pixels within the area of gamma index calculation was computed as the percentage of the maximal predicted dose.

### D. Trajectory log file

During each arc of VMAT delivery according to the QA plans or to actual treatments, the expected and actual position pairs of each TrueBeam machine axis, including leaf positions, gantry angle, and MUs, were logged every 20 ms up to 20 min and stored in a single binary file called the trajectory log file.^(10)^

For example, the positions of a certain leaf would be recorded as a trajectory of $\{e,{a\}}_{i}^{Tx},i=1,2,3,\ldots ,N_{\text{Tx}}$ (20 ms interval) during the treatment delivery, or $\{e,{a\}}_{i}^{QA},i=1,2,3,\ldots ,N_{\text{QA}}$ (20 ms interval) during the delivery of the QA plan, where, $e_{i}$ indicates the expected position that is ordered by the MLC controller at a certain time point, while $a_{i}$ indicates the actual position of the leaf at the same time point. *N* is the total number of snapshots, either for treatment or QA plan.

In this way, the expected and actual delivery parameters of each mechanical axis were easily extracted using an in‐house MATLAB (R2013a) tool. The data were analyzed and compared in terms of gantry motion and MLC leaf motion. A total of eight trajectory log files, acquired during the delivery of the four FF arc beams from the two QA plans and during the first treatment of the four FFF arc beams of the two treatment plans, were analyzed.

We categorized the differences into two groups. First, any difference in the planning stage can be evaluated through the differences in the expected positions of the FFF and FF beams. Second, the differences in the actual vs. expected positions of a FFF (or FF) beam will reflect the actual machine status. For example, since the dose rate of a FFF beam is twice higher than that of FF beam, it will be more challenging for the FFF beam to minimize the MU discrepancy.

### E. Film dosimetry

Verification using the trajectory log files can demonstrate the similarity of the QA plan to the original treatment plan, but these are complex deliveries with complex interactions of gantry speed, MLC speed, and dose rate. To further validate the method and strengthen the work, the comparison using film dosimetry was performed by delivering the original treatment plan and the corresponding QA plan to a custom‐made cylindrical acrylic phantom with embedded EBT3 film,^(11)^ which avoided the saturation limitation of the EPID. Film dosimetry was chosen to improve the spatial resolution, as these are SBRT beams. Relative gamma analysis was then used to compare the delivered FFF treatment plan to the delivered FF QA plan, as the absolute doses will be different with the reduction in MUs.

It should be noted that, for a good agreement in the direct dose comparison between the delivered FFF and FF beam, the dosimetric parameters of them are also the same in addition to the same beam delivery parameters. However, dose distributions of the treatment FFF plan and the corresponding QA FF plan are not the same because of the differences in dosimetric parameters, including depth dose and off‐axis beam profiles, off‐axis beam softening, output factors, and leakage under jaws. For example, the 10 cm percent depth dose of FFF beam is 2%–3% lower than the FF beam of the same nominal energy, and off‐axis factor of FFF beam is 80%–90% at 5 cm from the central axis due to absence of flattening filter.

Before the direct comparison of the delivered FFF film to the delivered FF film using gamma index, the dose‐to‐dose comparison between the FFF plan and FF plan was first performed to assess the magnitude of dose difference and its influence on the film‐to‐film comparison. It showed that the shape of dose profile was similar within 1%, except 2%–3% of the absolute dose level shift. It could be explained that, since the field size of the tested SBRT beams is as small as 4–5 cm, the profile difference is minimal inside such small fields and thus resulted in similar dose distributions between FFF and FF beams. However 2%–3% of absolute dose level shift was caused by 2%–3% difference in beam quality. Gamma passing rates with 2%/2 mm criteria were 89.3% to 100% in absolute dose comparison, but improved 99.6% to 100% in relative dose comparison. Since this absolute dose difference would affect in the film measurement while dose distributions are similar, applying relative dose comparison in gamma analysis between the delivered FFF and FF beam would eliminate this absolute dose difference and give good agreements if beam delivery machine parameters would be close to each other. The EBT3 films were scanned using an Epson 10000XL scanner (US Epson, Long Beach, CA) through the FilmQA Pro software (Ashland Advanced Materials, Niagara Falls, NY) and followed the procedure described by Lewis et al.^(12)^ All the irradiated film images were converted to dose map using a calibration response curve measured along with the experiment. The resulting dose map of delivered FFF beam was compared with the corresponding FF beam and evaluated using gamma analysis with dose tolerance of 2% within 2 mm.

## III. RESULTS

**Figure 2:**
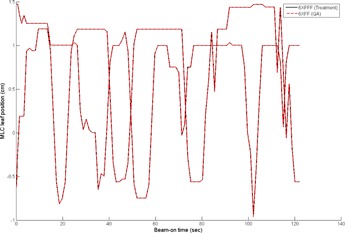
Comparison of the actual trajectories of the central leaf pair during the treatment of the 6XFFF clockwise (CW) arc beam and during delivery of the 6XFF CW arc beam of the corresponding QA plan (i.e., $\{{a_{i}^{Tx}\}}_{\text{MLC}}$ vs. $\{{a_{i}^{QA}\}}_{\text{MLC}}$). (See the text for notation.)

**Figure 3:**
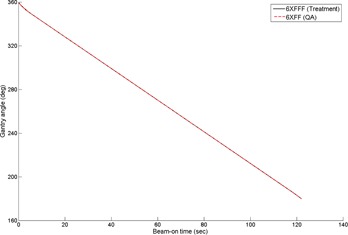
Comparison of the gantry motions for the treatment of the 6XFFF CW arc beam and corresponding 6XFF beam of the QA plan.

In our analysis of the trajectory log files, the motion of gantry rotation, MLC leaf positions, and delivered MUs were compared between the treatment and the QA plans. As an example, the actual trajectories of a central leaf pair during the treatment of the 6XFFF clockwise (CW) arc beam and during delivery of the 6XFF CW arc beam of the corresponding QA plan (i.e., $\{{a_{i}^{Tx}\}}_{\text{MLC}}$ vs. $\{{a_{i}^{QA}\}}_{\text{MLC}}$) are presented in Fig. 2. As expected, the leaf positions of the QA plan were the same as those of the treatment plan. Similarly, for the actual trajectories of the gantry rotation (i.e., $\{{a_{i}^{Tx}\}}_{\text{GA}}$ vs. $\{{a_{i}^{QA}\}}_{\text{GA}}$) as shown in Fig. 3, the gantry motions during treatment were the same as those of the delivery of the QA plan.

The mean and maximum differences of the expected positions between the treatment and the QA plans for the fractional MU, gantry angle, and 120 leaf motions (i.e., $\text{mean}\{e^{Tx}-{e^{QA}\}}_{i}$ and $\text{max}\{e^{Tx}-{e^{QA}\}}_{i}$) during delivery of the four arc beams are summarized in Table 1.

Overall, the maximum discrepancies of the expected positions between the treatment and QA plans were within 0.002 MU for the MU, 0.06° for the motion of gantry rotation, and 0.006 mm for the positions of the MLC leaves. This confirms that the two plans are exactly the same in terms of behavior of the MU delivery, gantry motion, and MLC leaf motions.

On the other hand, the mean and maximum differences in the actual positions against the expected positions for all time points are summarized in Table 2.

As for the MU, the mean and maximum discrepancies of the treatment FFF beams were within 0.04 and 0.2 MU, respectively, with two‐ to four‐fold larger than those of the corresponding QA FF beams. This is likely due to the higher dose rate of FFF beams. One exceptional large discrepancy was observed in the maximum difference in MU of the 10XFFF CW treatment beam, which will be addressed separately in the Discussion section below. In contrast, the maximum discrepancies in the gantry angle and leaf positions were within 0.02° and 0.07 mm, respectively, which were similar for both the treatment FFF beams and the QA FF beams.

Sun et al.^(10)^ reported similar results in an analysis of trajectory log files of 127 IMRT patients, in which the discrepancies of the actual positions against the expected positions were within 0.25 mm for multileaf collimator positions, 0.3° for gantry angles, and 0.13 MUs for MU delivery accuracy.

**Table 1:** Mean and maximum differences in the expected trajectories between the treatment and QA plans for the MU, gantry angle, and 120 leaf motions (i.e., $\text{mean}\{e^{Tx}-{e^{QA}\}}_{i}$ and $\text{max}\{e^{Tx}-{e^{QA}\}}_{i}$ during delivery of four arc beams (see the text for notation)

	*MU*		*No. Snapshots*		*Mean Differences* ^a^			*Maximum Differences* ^b^		
*Arc No*	*Treatment (FFF beam)*	*QA (FF beam)*	*Treatment(FFF beam)*	*QA (FF beam)*	*MU*	*Gantry Angle (deg)*	*Leaf Positions (mm)*	*MU* ^c^	*Gantry Angle (deg)*	*Leaf Positions (mm)*
6XFFF.CW	2434	1217	6103	6103	0.002	0.0003	0.00004	0.002	0.001	0.004
6XFFF.CCW	2446	1223	6135	6135	0	0.0003	0.00004	0	0.02	0.004
10XFFF.CW	1572	786	3953	3952	0	0.0006	0.0002	0	0.001	0.006
10XFFF.CCW	1572	786	3952	3952	0	0.0007	0.0002	0	0.06	0.006

a $\text{mean}\{e^{Tx}-{e^{QA}\}}_{i}$

b $\text{max}\{e^{Tx}-{e^{QA}\}}_{i}$

c max{eMUTx−eMUQA× MUtotalTx ∫∫/ MUtotalQA}i

CW=clockwise; CCW=counter clockwise.

**Table 2:** Mean and maximum differences in the actual positions against the expected positions for all time points (see the text for notation)

	*MU (Mean/Max)* ^a^		*Gantry Angle (deg) (Mean/Max)*		*Leaf Positions (mm)(Mean/Max)*	
*Arc No*.	*Treatment (FFF beam)*	*QA (FF beam)*	*Treatment (FFF beam)*	*QA (FF beam)*	*Treatment (FFF beam)*	*QA (FF beam)*
6XFFF.CW	0.02/0.13	0.02/0.04	0.05/0.21	0.07/0.21	0.008/0.05	0.008/0.05
6XFFF.CCW	0.02/0.11	0.02/0.04	0.03/0.17	0.02/0.17	0.007/0.04	0.007/0.05
10XFFF.CW	0.04/0.68	0.01/0.03	0.05/0.23	0.06/0.24	0.009/0.06	0.009/0.06
10XFFF.CCW	0.04/0.12	0.01/0.03	0.03/0.20	0.02/0.20	0.010/0.07	0.010/0.07

a $\text{mean}\{a^{Tx}-{e^{Tx}\}}_{i}$ and $\text{max}\{a^{Tx}-{e^{Tx}\}}_{i}$ for the treatment of FFF beams; similarly $\text{mean}\{a^{QA}-{e^{QA}\}}_{i}$ and $\text{max}\{a^{QA}-{e^{QA}\}}_{i}$ for the QA plan of FF beams.

As summarized in Tables 1 and 2, the discrepancies in the expected positions between the treatment and QA plans are roughly an order of magnitude smaller than the actual delivery accuracies, indicating the small discrepancies between the treatment and QA plans can be well ignored.

The total MU of the QA plan was set as an integer value close to the half of the total MU of the treatment plan. When the treatment MU is odd integer, the round‐off error might occur and add some discrepancies on the QA plan. A separate test was performed by modifying the total MU of the treatment plan as an odd integer to maximize the round‐off error. The trajectory log file of this plan compared to that of the QA plan showed that the figures would increase by a discrepancy of 1 MU, 0.2° for the motion of gantry rotation, and 0.7 mm in the positions of MLC leaves. The same difference would propagate into discrepancies in the actual positions between the treatment and QA plan.

The gamma passing rates and mean absolute dose difference of portal dosimetry for each arc beam are shown in Table 3. All gamma passing rates were above 96%, with the measured doses being 1.9%–2.3% higher than the predicted doses. Similar systematic deviations were observed for VMAT by Van Esch et al.^(4)^ and Vinall et al.,^(5)^ and can be explained by the slight nonlinear behavior of the portal dose acquisition; a larger acquisition time of VMAT delivery (more than 1 min) was seen compared to that of the normal dose calibration exposing only 100 MU at the dose rate of 300–600 MU/min. The authors suggested an exposure of 800 MU, instead of 100 MU, for the dose calibration for VMAT.

**Figure 4:**
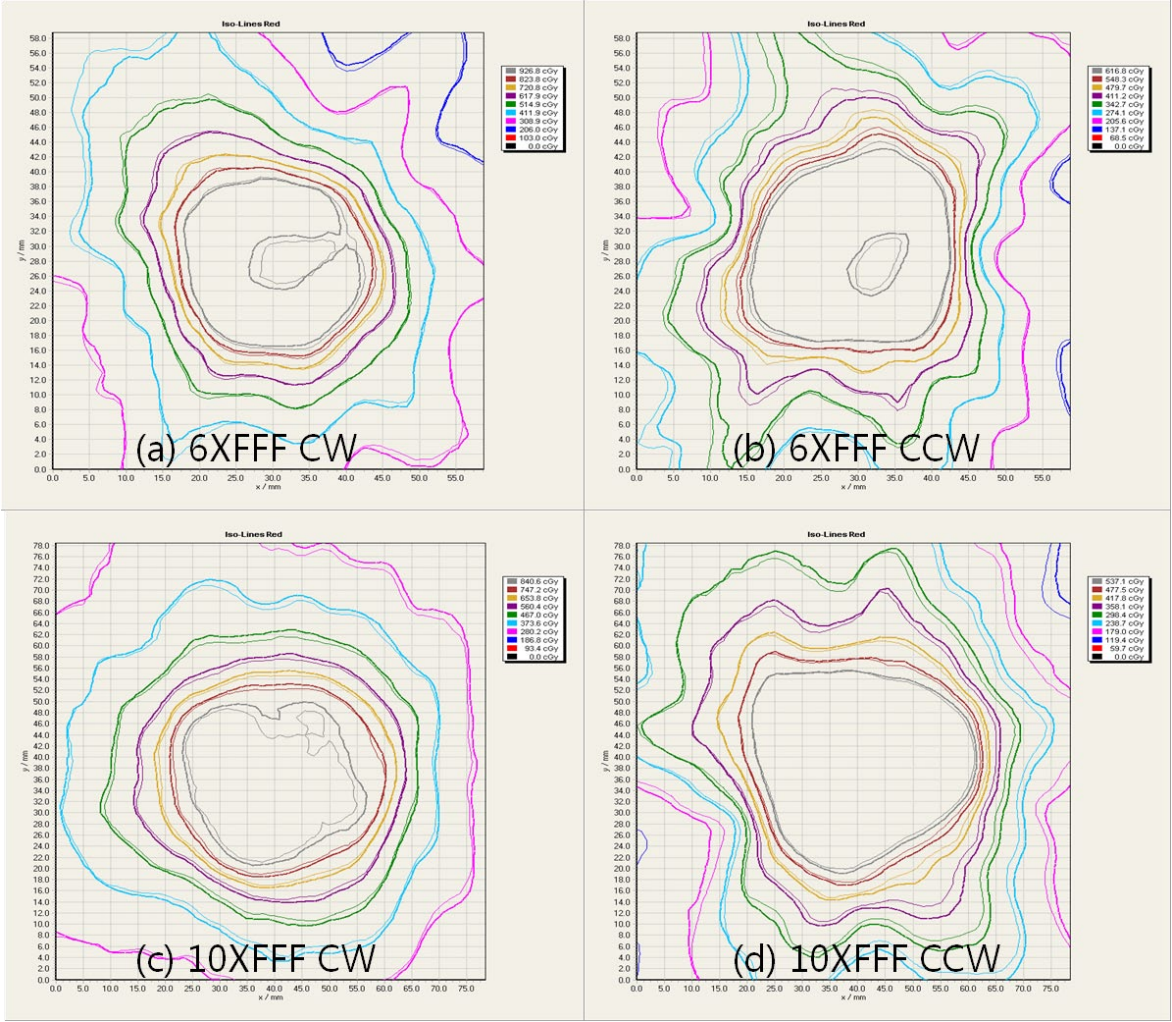
Comparisons of delivered dose distributions between the treatment FFF beam (thin line) and the corresponding QA FF beam (thick line): (a) 6XFFF CW, (b) 6XFFF CCW, (c) 10XFFF CW, and (d) 10XFFF CCW.

Figure 4 shows the isodose lines that directly compare the film measurements between the delivered treatment FFF and QA FF beams. As expected from the trajectory log files, the differences between the original treatment plan and the QA plan was minimal, and showed higher than 99.0% of gamma passing rate under 2%/2mm criteria, showing the difference is minimal, and also confirming the effectiveness of the proposed method.

**Table 3:** Passing rates of gamma index <1 and the mean absolute dose difference

*Arc No*.	*Gamma Passing Rate (%)*	*Mean Absolute Dose Difference (%)*
6XFFF.CW	99.2	2.3
6XFFF.CCW	99.3	2.0
10XFFF.CW	96.1	1.9
10XFFF.CCW	98.6	2.0

## IV. DISCUSSION

To resolve the current limitation of the portal dosimetry restricted to IMRT QA with FF beams, we proposed a practical approach that expands the current method applicable to IMRT QA with FFF beams.

In our suggested approach, the QA plan is not the same as the actual plan delivered to patients in terms of the energy, total MU, and dose rate. Therefore it may be argued that our proposed approach, which involves changing the treatment plan with the energy, dose rate, and MU, goes against the principle of verifying the delivered plan to the patient. We also carefully speculated various aspects where the proposed QA plan could miss the potential problems of the original treatment plan. They might be caused from the planning (dose calculation) side and the delivery (machine) side. The problem from the planning side would be mainly relevant with the accuracy of the TPS commissioning, including beam and MLC modeling. Assuming that the treatment planning system might be commissioned with similar level of uncertainty for all energies, the energy dependency could be ignored in the treatment planning side which computes the predicted dose images. Besides, checking the accuracies of the beam and MLC modeling should be done at the stage of the TPS commissioning, and thus not the purpose of the individual patient‐specific IMRT QA. On the other hand, our proposed method cannot identify energy‐specific potential errors that could occur in the machine side, such as variations in beam output or symmetry.

**Figure 5:**
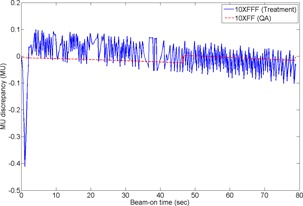
Actual delivered MU discrepancy against the expected values as a function of time for the 10XFFF CW beam of the treatment and the corresponding 10XFF CW beam of the QA plan. Note that the original data of 20 ms interval is resampled in 200 ms interval for illustration purpose. Due to wider dose‐rate modulation for the FFF beam, the MU discrepancy against the expected values fluctuated more frequently and widely compared to that of the FF beam. The increased discrepancy of the FFF beam during 1 sec from the beginning of beam delivery, which might be caused by dose rate instability during beam startup, was subsided shortly. In regard to the QA FF beam, the discrepancy of delivered MU against the expected values was seemingly corrected only at once about 45 sec when the discrepancy was increased above a certain preset tolerance limit.

One interesting finding, noticed from Table 2, is the minor dose‐rate instability of the 10XFFF CW beam at the beginning of beam delivery, as shown in Fig. 5, which resulted in an increased MU discrepancy at the beginning that quickly subsided. However, this pattern disappeared during the second beam delivery, indicating that this type of beam‐to‐beam variation is not energy‐specific.

As for the data transfer issue, since the QA plan is created by first copying the original treatment plan, if there were something wrong in the original plan such as leaf sequence data, then the QA plan would be also inherited the same problem. We believe the other way round would not occur. The main reason behind changing the energy in the QA plan was that the current version of TrueBeam intentionally prohibits the functionality of portal dosimetry for FFF beams due to the aforementioned saturation effect. This limitation can be easily overcome by manufacturer inclusion of FFF beams in the applicability of this tool.

Other energy‐relevant parameters would include the dosimetric leaf gap parameters and leaf transmission factors. Using the same methodology described by Wasbo and Valen,^(13)^ the measured leaf gap parameters of the TrueBeam HD120 MLC were 0.732 mm and 0.669 mm for 6XFF and 6XFFF, respectively, while they were 0.832 mm and 0.798 mm for 10XFF and 10XFFF, respectively. The leaf transmission factors of the TrueBeam HD120 MLC were measured as 0.012 and 0.010 for 6XFF and 6XFFF, respectively, and 0.014 and 0.012 for 10XFF and 10XFFF, respectively. These minor discrepancies due to small changes in beam quality indicate a minimal dosimetric effect of the proposed approach on IMRT QA.

By matching the speeds of gantry rotation and leaf motion with those of the treatment plan, the QA plan can detect errors that might occur due to mechanical issues.

Even after all the speculations we discussed, there are still concerns about the differences between the plan that is delivered for QA and the plan that is used for treatment. If the proposed technique is used as the only plan QA, the data integrity of the patient plan is not tested. In addition, beam‐specific modeling is not tested. The proposed approach would not detect errors in, for example, output factor configuration or MLC modeling. One could overcome this weakness by pairing the proposed method with an additional step, such as an ionization chamber measurement. We, therefore, propose an alternative, yet more complete, QA procedure as follows:

1. Ion‐chamber–based point dose measurement with the actual treatment plan.
2. Portal dosimetry with the QA plan derived from the proposed technique.
3. Comparison of the trajectory log files between the patient plan and the QA plan to ensure the differences are negligible.

Following the suggested procedure, we performed the point‐dose measurements for the treatment and QA plans with a CC13 ionization chamber and the same cylindrical phantom used for the film measurements, and the results are shown in Table 4. We also compared the trajectory log files of the treatment plans obtained during the point‐dose measurements with those of the QA plans recorded during the portal dosimetry, and confirmed that the results are similar as presented in the Tables 1 and 2 in the Results section.

**Table 4:** Comparisons between the plan and measured point doses with a CC13 ionization chamber and the same cylindrical phantom used for film dosimetry

	*Plan Dose (cGy)*		*Measured Dose (cGy)*		*Difference (%)* $(D_{\text{meas}}-D_{\text{plan}})/D_{\text{plan}}{\;}^{*}100$	
*Arc No*.	*Treatment (FFF beam)*	*QA (FF beam)*	*Treatment (FFF beam)*	*QA (FF beam)*	*Treatment (FFF beam)*	*QA (FF beam)*
6XFFF.CW	959.8	489.6	967.4	498.2	0.8	1.8
6XFFF.CCW	721.6	371.3	735.2	381.5	1.9	2.8
10XFFF.CW	897.4	458.2	890.8	455.9	−0.7	−0.5
10XFFF.CCW	578.4	298.4	588.9	304.3	1.8	2.0

## V. CONCLUSIONS

We introduce a simple method to expand a routinely used portal dosimetry for pretreatment IMRT QA to FFF beams. We believe that our proposed method is useful for IMRT verification QA, and can be used as an interim solution before more sophisticated and direct methods are developed.

## ACKNOWLEDGMENTS

This work was supported by Radiation Technology R&D program (2013M2A2A7043506) through the National Research Foundation of Korea funded by the Ministry of Science, ICT & Future Planning.
